# Challenges in reducing group B *Streptococcus* disease in African settings

**DOI:** 10.1136/archdischild-2016-311419

**Published:** 2016-10-18

**Authors:** Yo Nishihara, Ziyaad Dangor, Neil French, Shabir Madhi, Robert Heyderman

**Affiliations:** 1 Malawi-Liverpool-Wellcome Trust Clinical Research Programme, University of Malawi College of Medicine, Blantyre, Malawi; 2 Department of Paediatrics, Faculty of Health Sciences, University of the Witwatersrand, Johannesburg-Braamfontein, South Africa; 3 Department of Science and Technology/National Research Foundation: Vaccine Preventable Diseases, Faculty of Health Sciences, University of the Witwatersrand, Johannesburg-Braamfontein, South Africa; 4 Medical Research Council: Respiratory and Meningeal Pathogens Research Unit, Faculty of Health Sciences, University of the Witwatersrand, Johannesburg-Braamfontein, South Africa; 5 Institute of Infection and Global Health, University of Liverpool, Liverpool, UK; 6 Division of Infection and Immunity, University College London, London, UK

**Keywords:** group B streptococcus, neonatal sepsis, intrapartum antibiotic prophylaxis, GBS vaccine, Africa

## Abstract

Group B *Streptococcus* (GBS) is a leading cause of neonatal sepsis and meningitis in high-income settings and is associated with high rates of neonatal mortality and morbidity. There is now increasing evidence to suggest that there is a high GBS disease burden in resource-limited countries, and it is therefore critically important to identify suitable and practical preventive strategies. In Europe and North America, intrapartum antibiotic prophylaxis (IAP) has led to a dramatic reduction of early-onset GBS disease. However, the methods for identifying pregnant women who should receive IAP and how to reduce late-onset GBS disease are not without controversy and are challenging for most sub-Saharan African countries. GBS vaccines are approaching phase III trials but are still under development. This review aims to explore the current evidence related to strategies for reducing invasive GBS disease in an African setting, the development of a GBS vaccine and whether preventative measures against GBS disease can be practically implemented.

## Introduction

Worldwide, under-five mortality has fallen from 12.7 million in 1990 to 6.3 million in 2013.^1^ ^2^ However, progress needs to be made to reduce mortality in the first month of life, particularly in resource-limited settings where neonatal deaths accounted for 44% of all under-five mortality in 2013.^3^ In Africa, the neonatal mortality rate (31 per 1000 live births) is almost 4–5 times higher than that of the Americas (8 per 1000 live births) and Europe (6 per 1000 live births).^3^ Bacterial infection (sepsis, meningitis, pneumonia) is a leading cause of 2.9 million global neonatal deaths worldwide.^4^ Implementing strategies to reduce preventable infection-related neonatal deaths by 2030 to meet the WHO Sustainable Development Goal is a global health priority.

Group B *Streptococcus* (GBS) is a leading cause of neonatal sepsis and meningitis in high-income countries and is associated with high rates of mortality and morbidity.^5^ ^6^ There is increasing evidence that the burden of invasive GBS disease in low–middle-income settings is underappreciated, particularly in Africa.^7^ ^8^ Prevention of neonatal invasive GBS disease may therefore have a considerable impact on under-five mortality.

The use of intrapartum antibiotic prophylaxis (IAP) has been associated with an 80% reduction in early-onset GBS disease (EOD: 0–6 days of life);^9^ however, GBS remains a leading cause of severe neonatal infection. The ineffectiveness of IAP in protecting against late-onset disease (LOD: 7–89 days of life), combined with a high incidence of EOD in preterm babies (1.0 per 1000 live births), is a contributing factor.^9–11^ The evaluation of IAP as a strategy for the prevention of EOD has largely been undertaken in high-income settings,^12^ ^13^ and there is paucity of data on its suitability and practicality in resource-limited settings, where there are additional constraints, such as the lack of routine access to antenatal and intrapartum care, especially in rural settings. WHO predicts that the proportion of live births that occur in sub-Saharan Africa will continue to increase.^3^ Strategies to reduce the burden of invasive GBS disease and consequently under-five mortality in resource-limited settings is a public health priority.

In this review, we summarise the pathogenesis of GBS colonisation and explore the current evidence related to reducing invasive GBS disease, the development of a GBS vaccine and whether preventative measures against invasive GBS disease can be put into practice in resource-limited countries.

## Pathogenesis

GBS is a Gram-positive bacterium, and there are 10 serotypes based on their capsular polysaccharide composition (Ia, Ib, II–IX). The polysaccharide capsule is a major virulence factor contributing to bacterial evasion of phagocytic clearance.^10^ Serotype III is the most common invasive isolate and accounts for 30–50% of EOD and majority of LOD.^13^ ^14^ Other putative virulence factors include haemolysin, C5a peptidase and serine protease, but their role in disease causation remains to be determined.^15^

EOD occurs following acquisition of GBS by the fetus or newborn from their rectovaginally colonised mother. Though 30–70% of the newborns of GBS colonised women are themselves colonised at birth, only 1–3% go on to develop EOD.^12^ ^13^ ^16^ Infection of the fetus can occur either by invasion of amniotic fluid following prolonged rupture of membranes (PROM) or through direct acquisition during birth.^10^ GBS can invade through macroscopically intact membranes possibly through microtears of the amniotic sac.^17^ GBS β-haemolysin promotes lung epithelial cell invasion, leading to invasion into blood vessels.^6^ ^18^ Hence, inhalation of infected amniotic fluid or genital secretions can cause bloodstream invasion, sepsis and meningitis.^18^ Disease progression is rapid, with 90% of EOD presenting within 24 hours of delivery, and frequently already manifest at birth.^10^ This, along with the failure of chlorhexidine vaginal douches during labour to reduce EOD,^19^ suggests that most EODs commence in utero.

In contrast, the pathogenesis of LOD is uncertain, and the risk factors and mode of GBS acquisition by the neonate are less well defined.^20^ In LOD, GBS may be acquired horizontally via hospital, community or environmental contacts. Breast milk as a source of GBS in LOD has also been postulated.^21–24^ The role of maternal rectovaginal colonisation and whether there are other sources of GBS acquisition contributing to LOD require further study.^10^

Greater knowledge of the true rate of maternal GBS carriage and neonatal disease remains critical; in particular, the contribution of in utero GBS to both preterm labour and stillbirth will provide a broader insight into the total burden. Furthermore, the disease-causing serotypes in different resource-limited settings need to be investigated, and new whole-genome sequencing approaches applied to investigate the relationship between invasive GBS and carriage isolates.^10^

## Maternal carriage of GBS and the impact of HIV

The main reservoir for GBS is in the large intestine and the lower genital tract.^25^ Rectovaginal colonisation by GBS is dynamic and can be transient, intermittent or persistent.^26^ Certain factors also favour the transmission of GBS from mother to infant, including maternal colonisation, density of colonisation and PROM.^10^ GBS bacteriuria (found in 2–7% of pregnant women)^27^ ^28^ is an indicator for heavy genital tract colonisation and is associated with a higher risk of chorioamnionitis, EOD and LOD.^29^ In addition, virulence of the organism, particularly of the ST17 genotype, may favour transmission.^10^ ^14^ ^30^ Factors that appear to reduce transmission and/or invasive disease are exposure to intrapartum antibiotics, elective caesarean section in the absence of labour and high maternal GBS serotype-specific capsular antibodies.^11^ ^31^

Studies based upon vaginal-rectal swabs both in both high-income and low–middle-income countries (LMICs) have shown that 10–40% of women are rectovaginally colonised by GBS during pregnancy.^10^ A review of GBS carriage by Stoll *et al*^32^ in low-income countries showed 19% GBS carriage in sub-Saharan African women, while country-specific studies in Africa showed carriage rates of 28.4% in South Africa,^26^ 21.2% in Malawi,^33^ 23% in Tanzania^34^ and 22% in the Gambia.^35^

Although maternal HIV infection has not been associated with higher prevalence of GBS colonisation,^36^ a study in Malawi showed a direct relationship between CD4+ count and GBS carriage, with the highest GBS carriage prevalence in HIV-infected women with CD4 count >500 cells/mm^3^.^33^ Reduced GBS carriage at lower CD4 counts may be due to the competitive exclusion of GBS by ecological changes in the microbial flora and the increased presence of bacterial vaginosis and anaerobes.^33^ Natural maternal capsular and protein antibody concentrations against GBS were found to be lower in HIV-infected South African women, which may also increase the vulnerability of HIV-exposed infants to GBS EOD and LOD.^37^ This has been reflected in invasive GBS disease incidence in South Africa, which showed a 2.25-fold greater incidence in HIV-exposed compared with HIV-unexposed infants.^36^

## Intravenous intrapartum antibiotic strategy to prevent early-onset GBS disease

As neonates with GBS EOD are often already septic at birth, with rapid progression of disease, a strong focus has been placed on a secondary preventative strategy to treat at-risk mothers in labour.^10^

### Mechanism of action of IAP

Given that maternal rectovaginal colonisation is associated with EOD, IAP aims to reduce the vertical transmission of GBS from mother to baby by decreasing the colony count at time of delivery, and also to achieve an effective level of antibiotic in the fetal circulation.^38^ Achieving adequate level of antibiotic in the amniotic fluid may directly protect the neonate against EOD by preventing the proliferation of GBS in utero.^38^ ^39^ Clinical trials in the 1980s demonstrated that IAP reduced the incidence of early neonatal GBS disease and has formed the basis of IAP strategy.^40^ ^41^ A Cochrane review highlighted a statistically significant reduction in the early-onset neonatal GBS disease following IAP compared with no treatment, where in industrialised countries the number needed to treat to benefit was 25 (95% CI 14 to 100).^42^

### Choice of antibiotic and duration of administration

Globally, GBS remains fully susceptible to β-lactams,^43^ with benzylpenicillin^10^ or ampicillin^6^ most commonly administered. Erythromycin and vancomycin have been used; however, their efficacy has not been measured in controlled trials, and the ability of these antibiotics to achieve bactericidal levels in fetal circulation and amniotic fluid is uncertain.^6^ ^44–46^

The optimal duration of IAP to reduce vertical GBS transmission has also been debated.^6^ While the Center for Disease Control (CDC) recommends a minimum duration of 4 hours between IAP administration and delivery,^6^ ^47^ several studies have suggested that the duration of antibiotics may be reduced from 4 hours to 1–2 hours prior to delivery.^38^ ^48^ ^49^ Indeed, benzylpenicillin levels in cord blood have been shown to exceed the minimum inhibitory concentration for GBS as early as 1 hour after maternal administration.^38^

### Identifying those women who require IAP

Two common approaches are used to identify pregnant women who should receive IAP: universal screening and a risk-based strategy. More recently, rapid diagnostic tests have been developed.

#### Universal screening strategy

All pregnant women in the third trimester of pregnancy (ideally 35–37 weeks gestation) are offered screening for GBS colonisation. This method is recommended by the CDC and is practised widely throughout the USA. Those identified as GBS carriers are treated with antibiotics during labour. Women with GBS bacteriuria during the current pregnancy or a previous infant with invasive GBS disease also receive IAP, without the need for screening.^6^ As a result, the incidence of GBS EOD has declined dramatically, from 1.7 cases per 1000 live births in the early 1990s^6^ to 0.27 cases per 1000 live births by 2014.^50^

#### Risk-based approach

Several risk factors are associated with increased risk of EOD in the newborn, including preterm labour, PROM >18 hours, intrapartum fever >38°C, GBS bacteriuria in the mothers during the current pregnancy and previous sibling with invasive GBS disease.^51^ Several countries adopt a strategy to implement IAP when some or all of these risks are identified.

The UK Royal College of Obstetricians and Gynaecologists and the National Institute for Health and Care Excellence do not recommend routine bacteriological screening of all pregnant women,^11^ although if GBS is detected on an incidental vaginal swab or urine culture then IAP is recommended.^11^ This reflects the view that the incidence of EOD will not reduce any further with the introduction of universal screening for GBS in pregnancy; the incidence of EOD in the UK under the risk-based approach is 0.5/1000, which is similar to that of the USA, which employs universal screening.^11^ ^51^ Recently published surveillance data, however, indicate a small but possible rise in the incidence of EOD (per 1000 live births) in European countries, including from 0.30 in 1991 to 0.41 in 2010 in the UK,^52^ and from 0.11 in 1987 to 0.19 in 2011 in the Netherlands.^53^

#### Rapid diagnostic testing during labour

More recently, novel rapid methods to augment or replace traditional microbiological methods to identify GBS have emerged. Rapid real-time PCR tests demonstrated sensitivity from 62.5% to 100% and specificity from 84.6% to 100% compared with enriched GBS cultures.^51^ Di Renzo *et al*^51^ argue that this method could prove superior to risk-based approach or universal screening if undertaken when a woman is in labour as those who are current carriers of GBS are at most risk of passing GBS to their babies will be identified. Direct antigen detection tests are rapid and inexpensive, but not sufficiently sensitive for diagnosis.^51^ The loop-mediated isothermal amplification method to identify GBS could combine many of the advantages of PCR tests with the relative simplicity of antigen detection tests;^54^ however, this is still in development. In resource-limited settings, these tests at present may prove too expensive and logistically challenging as an alternative strategy in high-volume settings.

## Other approaches to reducing GBS-associated morbidity and mortality

### Early recognition of neonatal sepsis

Administration of IAP does not eradicate the risk of early-onset GBS disease. Furthermore, delay in administering IAP due to factors such as late presentation to the labour ward, rapid delivery or misidentification of risk factors/maternal GBS colonisation status can result in inadequate IAP cover. Early recognition of the signs of neonatal sepsis and commencing treatment promptly are crucial in preventing rapid invasive GBS disease progression. Further studies are needed in LMIC settings.

### Alternative routes of intrapartum antibiotic delivery

A randomised controlled trial to evaluate outpatient prenatal oral amoxicillin versus placebo did not significantly impact on GBS vaginal colonisation at the time of delivery (p=0.20).^55^ Few studies have evaluated the pharmacokinetics or effectiveness of IAP delivered by alternative routes, such as orally or intramuscularly. There could be a more feasible way to deliver intrapartum antibiotics to at-risk women in labour outside of a tertiary hospital setting in resource-limited countries and require further evaluation.

### Chlorhexidine

Chlorhexidine is a powerful mucous membrane disinfectant that can result in suppression of maternal GBS, and therefore has been proposed as a low-cost, easy-to-administer strategy in reducing neonatal GBS sepsis. However, in a recent Cochrane review of four trials of vaginal chlorhexidine there was no reduction of EOD (sepsis and/or meningitis) or GBS pneumonia, although it may reduce the GBS neonatal colonisation.^19^

### GBS vaccine

A GBS vaccine, administered to mothers in pregnancy, could provide protection to neonates against EOD and LOD via transplacental IgG antibody transfer to the fetus.^16^

Maternal vaccine-induced antibodies are transferred via the placenta to the fetus through an active transfer process in the second trimester of gestation onwards, providing protection to the newborn for the first few months of life.^56^ Transplacental antibodies, as well as breast milk antibodies, may therefore reduce both EOD and LOD.^16^ ^22^ By augmenting pre-existing maternal antibody levels, vaccination may also have the potential to prevent maternal intra-amniotic infections during pregnancy and postpartum endometritis,^57^ which could theoretically impact against GBS-associated premature delivery and stillbirths.^58^ However, in high HIV prevalence settings, the lower immunogenicity of a trivalent polysaccharide–protein conjugate vaccines in HIV-infected mothers (leading to lower transplacental antibody transfer) highlights the possible need for alternate dosing schedules.^59^ ^60^ A trivalent GBS polysaccharide–protein conjugate vaccine has undergone phase II trials in South Africa and Malawi, and has the potential to offer cost-effective protection against GBS sepsis.^59^

## Can GBS preventative measures be put into practice in a resource-limited setting?

The most promising strategy in reducing neonatal invasive GBS disease is maternal vaccination; first, it has the potential to prevent both EOD and LOD; second, it could be made to be cost-effective; and third, it may reduce GBS-related stillbirths and premature delivery. However, the vaccine is still in development, and it may be 10–15 years before it can be introduced into routine care. Meanwhile, strategies to identify at-risk pregnant women and to implement IAP in resource-limited settings need to be explored (table 1).

**Table 1:** Advantages and disadvantages of group B *Streptococcus* (GBS) carriage identification methods

	Universal screening	Risk-based approach	RDT
Advantages	Targeted prevention of GBS transmission from mother to baby Can monitor GBS carriage among women over time	Easier to implement—no laboratory set-up required No requirement for mass antenatal screening Cheaper to implement than universal screening or RDTs	Potential to rapidly identify those at highest risk of passing GBS to neonate and can be done intrapartum No requirement for mass antenatal screening Could be employed in preterm deliveries.
Disadvantages	Logistical challenges—relies on full laboratory set-up, appropriate transport and storage conditions and timely communication to clinical staff/pregnant women Difficult to collect specimens at correct gestation in areas where antenatal scans are not readily available Results can take 18–48 hours—results may not be available in time GBS colonisation state is dynamic, and GBS status may change from screening to delivery Will miss 7–11% of preterm deliveries, which can account for 32–38% of neonatal GBS EOD	Overlooks the biggest risk of GBS EOD, which is presence of maternal GBS colonisation or GBS bacteriuria Potential to overtreat pregnant women with IAP who do not carry GBS and miss those who may actually have GBS colonisation Will not prevent EOD in settings where there are more home deliveries or in primary healthcare settings with limited diagnostic and treatment facilities No samples collected—difficult to monitor true effect on GBS carriage rate, transmission and any development of antibiotic resistance	Cost issues: running the tests, storing of reagents, training of staff in performing tests and reading results Clinical relevance of molecular assay technique still needs to be quantified

EOD, early-onset GBS disease; IAP, intrapartum antibiotic prophylaxis; RDT, rapid diagnostic tests.

The first step would be to consider either a universal screening or a risk-based approach. Both strategies ultimately rely on the dependable availability of intravenous antibiotics and adequately trained medical staff. IAP may be possible to implement in a tertiary or district hospital setting, but will prove very difficult at a primary healthcare setting, where diagnostic and treatment facilities are limited, or in areas of high rates of home births. These limitations may result in a less dramatic reduction in EOD compared with high-income countries.

The advantage of universal screening at 35–37 weeks gestation is that it could identify those women with the highest risk of invasive GBS disease, (ie, GBS colonisation near time of delivery) and allow targeted treatment of GBS carriers with IAP. However, there are logistical difficulties in implementing this strategy in the resource-limited setting where antenatal care and laboratory facilities may be sparse or lacking. Accurately targeting women at 35–37 weeks gestation is a challenge where routine antenatal gestation calculation by ultrasound is not available. The transport, storage and processing of numerous specimens, and communication of results to the pregnant women or health professionals may be problematic.

On the other hand, a risk-based approach avoids mass screening, is cheaper to implement and could potentially achieve similar reductions in EOD compared with universal screening. Decision to treat can be made on admission, and therefore, laboratory support is not required. However, this approach potentially overlooks the main risk factor for EOD, which is maternal GBS colonisation/bacteriuria. Up to 50% of women with risk factors are not colonised with GBS,^10^ leading to unnecessary administration of antibiotics, while women with no risk factors in labour can still have babies with GBS disease.

**Figure 1:**
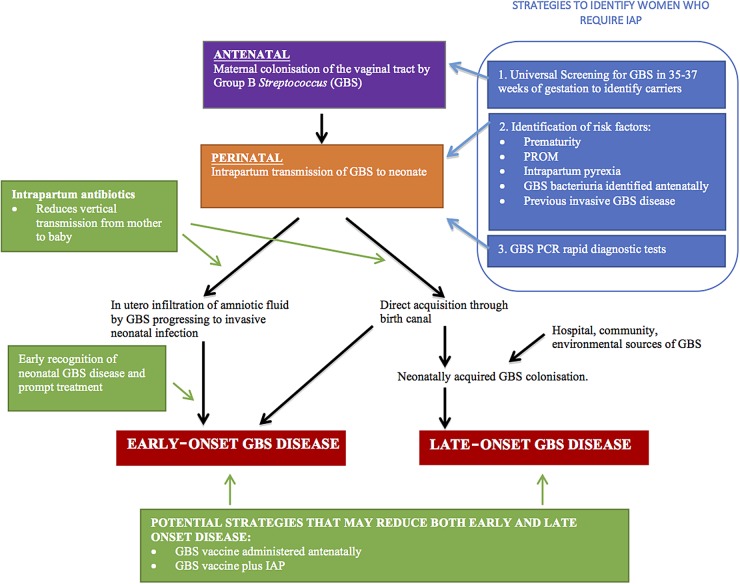
Pathogenesis of neonatal group B *Streptococcus* (GBS) disease and target for intervention. IAP, intrapartum antibiotic prophylaxis; PROM, prolonged rupture of membrane.

Intrapartum GBS screening with rapid real-time PCR testing was recommended by the Consensus Conference on GBS in Europe in 2014.^51^ However, the cost to implement and requirements for appropriate reagent storage and supply would make this a less attractive and practical alternative in the resource-poor setting. A simple, cheap point-of-care test could improve the timely implementation of IAP (figure 1).

A possible variation to the use of IAP is to revisit the efficacy of maternal intramuscular and high-dose oral antibiotics in labour, which potentially circumvents the problem of delivering intravenous antibiotics in primary healthcare or home settings. The pharmacokinetics and drug concentration in utero need to be further evaluated, and ultimately, if promising, these need to be tested in controlled trials.

## Conclusion

The development of measures to reduce the infectious causes of neonatal mortality is critical, and supportive evidence is urgently needed. Although vaccine development appears to be the most feasible and cost-effective strategy to achieve long-term solution, this may take several years to come to fruition. Clinicians need to be able to recognise the early signs of neonatal sepsis and start the appropriate treatment promptly. Novel, affordable alternative approaches to prevention should be explored in parallel. Pharmacokinetics and pharmacodynamics in utero of high-dose oral intramuscular antibiotics could be revisited. Finally, critical barriers need to be addressed in selecting which prevention strategy is most suitable for the target population and cost-effectiveness evaluated to reflect the available healthcare infrastructure. Once introduced into policy, it will be essential that the appropriate surveillance is in place to monitor uptake, and impact on neonatal mortality and antimicrobial resistance.
